# Advancements in the diagnosis and management of premature ventricular contractions in pediatric patients

**DOI:** 10.3389/fped.2024.1373772

**Published:** 2024-03-20

**Authors:** Wenjing Zhu, Hui Yuan, Jianli Lv

**Affiliations:** ^1^ Department of Pulmonary and Critical Care Medicine, Shandong Provincial Hospital Affiliated to Shandong First Medical University, Shandong Provincial Clinical Research Center for Children's Health and Disease Office, Jinan, Shandong, China; ^2^ Department of Pediatric Cardiology, Shandong Provincial Hospital Affiliated to Shandong First Medical University, Shandong Provincial Clinical Research Center for Children's Health and Disease Office, Jinan, Shandong, China

**Keywords:** premature ventricular contractions, arrhythmia, diagnosis, management, children

## Abstract

**Background:**

Premature ventricular contractions (PVCs) are relatively common arrhythmias in the pediatric population, with implications that range from benign to potentially life-threatening. The management of PVCs in children poses unique challenges, and recent advancements in diagnostic and therapeutic options call for a comprehensive review of current practices.

**Methods:**

This review synthesizes the latest literature on pediatric PVCs, focusing on publications from the past decade. We evaluate studies addressing the epidemiology, pathophysiology, diagnosis, and treatment of PVCs in children, including pharmacological, non-pharmacological, and invasive strategies.

**Results:**

The review identifies key advancements in the non-invasive detection of PVCs, the growing understanding of their genetic underpinnings, and the evolving landscape of management options. We discuss the clinical decision-making process, considering the variable significance of PVCs in different pediatric patient subgroups, and highlight the importance of individualized care. Current guidelines and consensus statements are examined, and areas of controversy or limited evidence are identified.

**Conclusions:**

Our review underscores the need for a nuanced approach to PVCs in children, integrating the latest diagnostic techniques with a tailored therapeutic strategy. We call for further research into long-term outcomes and the development of risk stratification tools to guide treatment. The potential of emerging technologies and the importance of multidisciplinary care are also emphasized to improve prognoses for pediatric patients with PVCs.

## Introduction

Premature ventricular contractions (PVCs) constitute a common pediatric cardiac arrhythmia, characterized by ectopic depolarizations originating from the ventricular myocardium prior to a sinus beat. While PVCs can manifest as an isolated phenomenon in otherwise healthy pediatric hearts, their presence may also be indicative of underlying myocardial pathology or ion channel dysfunction (1, 2). The clinical ramifications of PVCs in pediatric cohorts are varied, ranging from benign incidental discovery to associations with increased morbidity, including the potential for cardiomyopathy, and in rare instances, sudden cardiac death (SCD) (3).

The pathogenesis of PVCs is multifactorial, encompassing enhanced automaticity, reentrant circuits, and triggered activity due to afterdepolarizations. Both early and delayed afterdepolarizations have been implicated in the genesis of PVCs, with the latter often associated with conditions that prolong repolarization, such as long QT syndromes (4). Additionally, the pediatric substrate often presents unique structural and electrophysiological considerations, including congenital heart disease (CHD) and primary arrhythmia syndromes, which may predispose to arrhythmogenic foci (5–7).

Recent advances in molecular genetics have underscored the contribution of genetic polymorphisms and mutations to the etiology of PVCs, particularly within the context of inherited arrhythmia syndromes. The identification of genotype-phenotype correlations has not only enhanced our understanding of these arrhythmias but has also opened avenues for targeted gene therapies (8). The diagnostic algorithm for PVCs in children integrates non-invasive modalities such as 12-lead electrocardiography, Holter monitoring, and advanced imaging, with invasive electrophysiological studies reserved for select cases (9).

Therapeutic interventions for pediatric PVCs are predicated on the stratification of arrhythmic risk, symptomatology, and the potential for hemodynamic compromise. The therapeutic armamentarium includes conservative management, antiarrhythmic pharmacotherapy, and interventional electrophysiology, such as radiofrequency catheter ablation, which has demonstrated increasing efficacy and safety in the pediatric demographic (5, 10). Despite these advances, the management of PVCs in children remains a topic of debate, with a paucity of pediatric-specific data necessitating extrapolation from adult literature and reliance on consensus guidelines (11, 12).

This review endeavors to elucidate the current state of knowledge on the epidemiology, pathophysiological mechanisms, diagnostic strategies, and management of PVCs in the pediatric population. By integrating contemporary research findings with clinical expertise, we aim to distill evidence-based recommendations for the management of this heterogeneous arrhythmia and propose future directions for research to fill the existing gaps in pediatric cardiology.

## Epidemiology

The epidemiological landscape of PVCs in the pediatric population is a reflection of their multifaceted origins, ranging from idiopathic presentations to associations with more complex cardiac anomalies (13). Frequent idiopathic PVCs and asymptomatic ventricular tachycardias in children and young adults are rare, especially in the first decade. In older children and young adults, the incidence increases, although exact numbers are unknown since most patients are asymptomatic (14). Pediatric PVCs exhibit variability in terms of frequency, complexity, and configuration, which can change over time and may be influenced by factors such as age, autonomic tone, and myocardial substrate (15). Holter monitoring studies have demonstrated a higher prevalence, detecting PVCs in up to 41% of children over a 24-hour period, suggesting that intermittent PVCs may be a common and transient occurrence in this demographic (16).

Among the pediatric age groups, adolescents frequently present with PVCs, which in the absence of cardiac abnormalities, are often deemed benign. However, the risk of PVC-associated cardiomyopathy necessitates longitudinal surveillance, particularly in the presence of high PVC burdens or symptomatic episodes (17, 18). Interestingly, a retrospective analysis in asymptomatic pediatric patients with structurally normal hearts has shown that there is no significant correlation between the burden of PVCs and left ventricular systolic function, suggesting that a high PVC burden alone may not be indicative of cardiac dysfunction in this demographic (19). The incidence of PVCs is also well known to increase with the presence of underlying CHD, with certain CHD subtypes such as tetralogy of Fallot and post-operative scar-related circuits predisposing to ventricular arrhythmias (20).

The role of gender in the epidemiology of pediatric PVCs remains an area of investigation. Some studies suggest a male predominance in arrhythmic presentations (21), while others have found no significant gender disparity (22). The impact of race and ethnicity on PVC prevalence and outcomes in children has not been reported, but studies in adults suggest a relationship. For instance, a cross-sectional analysis from the Atherosclerosis Risk In Communities (ARIC) study revealed that PVC prevalence on a 2-minute electrocardiogram (ECG) in middle-aged adults is influenced by factors including age, heart disease, sinus rates, ethnicity, sex, educational attainment, and electrolyte levels, with hypertension independently associated with a 23% increase in PVC prevalence, highlighting potential disparities and risk factors that may also be relevant in pediatric populations (23).

Family history and genetic predisposition play a critical role in the epidemiology of PVCs. Familial clustering of ventricular arrhythmias and the identification of causative mutations in genes encoding cardiac ion channels and structural proteins emphasize the importance of genetic counseling and family screening in affected individuals (24).

## Pathophysiology

The pathophysiological underpinnings of PVCs in children are intricate, reflecting a diverse spectrum of cellular and molecular mechanisms (25–27). At the cellular level, PVCs originate from abnormal electrical activity within the ventricular myocardium, which can be classified into three primary categories: enhanced automaticity, triggered activity, and reentry.

Enhanced automaticity refers to the spontaneous generation of action potentials from ectopic pacemaker cells within the ventricles, which can be facilitated by modifications in the transmembrane ion gradients or increased sympathetic tone (15). This phenomenon may be exacerbated in the presence of myocardial injury or ischemia, where damaged cells may display heightened sensitivity to catecholaminergic stimulation. A study examining pediatric patients with left ventricular noncompaction (LVNC) further elucidates this relationship, revealing that those with normal or mildly impaired ventricular function generally have favorable outcomes, yet exhibit an increased incidence of simple ventricular ectopy, particularly as systolic function worsens. This suggests a potential link between myocardial damage and arrhythmic burden (28).

Triggered activity arises from afterdepolarizations, which can be early (EADs) or delayed (DADs) relative to the action potential. EADs typically occur during the repolarization phase of the cardiac cycle and are associated with conditions that prolong the action potential duration, such as acquired or congenital long QT syndromes (29). Conversely, DADs manifest after the completion of repolarization and are often linked to intracellular calcium overload, a common feature in catecholaminergic polymorphic ventricular tachycardia (CPVT) (30, 31).

Reentrant circuits, the third mechanistic category, are facilitated by the presence of heterogenous conduction velocities within the ventricular myocardium, creating a substrate for the perpetuation of abnormal electrical loops. This can occur in the context of structural heart disease, where fibrotic or scarred tissue from surgical repair or myocarditis provides a non-conductive barrier that disrupts the normal propagation of electrical impulses (20).

Genetic predispositions also play a critical role in the pathophysiology of pediatric PVCs, with several ion channelopathies being implicated in arrhythmogenesis. Mutations in genes encoding for cardiac sodium (SCN5A), potassium (KCNQ1, KCNH2), and calcium (CACNA1C) ion channels can result in dysfunctional ion transport, directly affecting the electrophysiological properties of cardiomyocytes (27). Additionally, structural protein mutations, such as those found in arrhythmogenic right ventricular cardiomyopathy (ARVC), can lead to mechanical and electrical instability of the cardiac tissue (32). Complementing these findings, the International Triadin Knockout Syndrome Registry has revealed that recessive null mutations in TRDN-encoded cardiac triadin contribute to severe arrhythmic conditions, including exercise-induced cardiac arrest in young children, which are refractory to conventional therapies and often necessitate implantable defibrillators due to recurrent ventricular arrhythmias (33). These studies emphasize the importance of genetic testing for specific mutations, such as those in TRDN, which may account for otherwise unexplained cardiac events in the pediatric population.

Moreover, neurohormonal influences, particularly the modulation of the autonomic nervous system, have been observed to affect the incidence and severity of PVCs. An increase in sympathetic activity or a decrease in parasympathetic tone can precipitate PVCs by altering the electrophysiological state of the ventricular myocardium, highlighting the importance of the autonomic nervous system in pediatric arrhythmias (34).

Understanding the pathophysiology of PVCs is essential for informing the development of targeted therapies and risk stratification strategies in the pediatric population. Future research is required to unravel the complex interplay between genetic factors, autonomic regulation, and myocardial substrate that contributes to the manifestation of PVCs in children.

## Diagnosis

The diagnosis of PVCs in the pediatric population is a multi-step process that begins with a thorough history and physical examination and is followed by various non-invasive and, occasionally, invasive diagnostic modalities. The initial clinical assessment aims to determine the presence of symptoms such as palpitations, dizziness, or syncope, which may be suggestive of PVCs or more significant arrhythmias (22). A detailed family history is also essential to identify potential inheritable syndromes associated with arrhythmogenic risk.

The cornerstone of PVC diagnosis is the 12-lead ECG, which provides information on the morphology, frequency, and pattern of PVCs. Characteristic ECG findings include a premature QRS complex with a duration typically greater than 120 ms, an abnormal QRS axis, and the absence of a preceding P wave. The compensatory pause following the PVC, a result of the resetting of the sinus node, is a further diagnostic criterion.

When PVCs are infrequent or not captured on a standard ECG, ambulatory Holter monitoring or event recording may be employed. These extended ECG recording techniques can quantify PVC burden, categorize complexity, and document any associated symptoms. Holter monitoring is particularly useful for evaluating the circadian variation of PVCs and their relationship with exercise and sleep (3).

Exercise stress testing is another diagnostic tool that can elucidate the behavior of PVCs during physical exertion. Typically, benign PVCs tend to decrease in frequency with increased heart rate during exercise, while PVCs due to underlying pathology may persist or increase (35). Additionally, exercise testing can identify exercise-induced arrhythmias and assess functional capacity and hemodynamic response in pediatric patients (35, 36).

Furthermore, a recent study has demonstrated the effectiveness of mobile cardiac outpatient telemetry (MCOT) in the pediatric population, achieving a diagnostic rate of 61% for suspected arrhythmias, outperforming traditional event and Holter monitors, with minimal complications (37).

Echocardiography is a fundamental non-invasive imaging modality that provides information on cardiac structure and function, which is vital for ruling out structural heart disease as a cause of PVCs. The presence of ventricular dilation, dysfunction, or other abnormalities may guide further investigation and management (18, 38).

In cases where the etiology of PVCs remains uncertain, especially in the presence of a high arrhythmic burden or failed antiarrhythmic therapy, cardiac magnetic resonance imaging (CMR) can be utilized. CMR offers detailed tissue characterization and can detect myocardial fibrosis or fatty infiltration, as seen in conditions such as ARVC (39, 40).

For a subset of patients in whom invasive evaluation is warranted, an electrophysiological study (EPS) may be performed. EPS can delineate the precise origin of PVCs and assess the inducibility of sustained ventricular tachycardia, providing critical information for risk stratification and guiding therapeutic interventions such as catheter ablation (5).

Recent advances in genetic testing have also become an integral part of the diagnostic workup for PVCs, particularly in patients with a suspected inheritable syndrome or a family history of sudden cardiac death. Genetic testing can identify mutations associated with channelopathies or cardiomyopathies, facilitating personalized management strategies for affected individuals (41).

The diagnostic approach to PVCs in children is comprehensive, leveraging a combination of clinical assessment, non-invasive testing, and invasive procedures when indicated. Each diagnostic step is tailored to the individual patient, with the ultimate goal of identifying the underlying cause and determining the appropriate management strategy.

## Treatment

The therapeutic approach to pediatric patients with PVCs is tailored to the severity of symptoms, the underlying etiology, and the potential for adverse outcomes. Management strategies encompass a spectrum ranging from conservative observation to pharmacological intervention and, in select cases, invasive procedures.

### Non-pharmacological treatment

Non-pharmacological interventions, including lifestyle modifications, are recommended, particularly in the presence of modifiable risk factors. These modifications may involve the avoidance of stimulants such as caffeine and illicit drugs, ensuring adequate hydration, and addressing any electrolyte imbalances (42). Stress reduction techniques and biofeedback can also be beneficial in managing PVCs, especially when there is a clear association between stress and arrhythmic episodes (43).

### Pharmacological treatment

Pharmacological therapy is considered in pediatric patients who are symptomatic or when PVCs are associated with ventricular dysfunction or other forms of structural heart disease (17). Beta-blockers are often the first-line agents, particularly in cases where adrenergic stimulation is presumed to contribute to arrhythmogenesis. These agents serve to decrease myocardial oxygen demand, reduce sympathetic drive, and suppress ectopic ventricular activity (30). In cases refractory to beta-blockade or when beta-blockers are contraindicated, calcium channel blockers such as verapamil can be employed, particularly for idiopathic PVCs originating from His-Purkinje system and the outflow tract (44). Propafenone has been shown to be effective in suppressing PVCs, ventricular couplets, and nonsustained ventricular tachycardia, with a significant proportion of patients achieving suppression of arrhythmias (45). However, the long-term efficacy and safety of propafenone require careful consideration, as treatment response and patient compliance can vary. Antiarrhythmic medications, including Class I agents like flecainide, ivabradine, or Class III agents such as amiodarone, may be reserved for more complex or therapy-resistant PVCs due to their proarrhythmic potential and side effect profile (46, 47). The use of these agents necessitates close monitoring for efficacy and toxicity.

### Invasive strategies

Catheter ablation is an invasive strategy that is increasingly utilized in the pediatric population for the treatment of symptomatic, frequent, or hemodynamically significant PVCs that are unresponsive to medical therapy. This procedure involves the delivery of radiofrequency energy or cryoablation to the site of the ectopic focus, thereby eliminating the arrhythmogenic substrate (5, 17). Advances in mapping technologies have enhanced the safety and efficacy of catheter ablation, with success rates improving and complication rates decreasing (10, 48).

In patients with structural heart disease or those at high risk for sudden cardiac death, the implantable cardioverter defibrillator (ICD) may be indicated. However, the decision to implant an ICD in a pediatric patient requires careful consideration due to the lifelong implications, potential need for lead revisions, and the psychological impact on the patient (49).

The treatment of PVCs in children is complex and requires individualized decision-making. The risk-benefit profile of each intervention must be carefully weighed, taking into account the patient's clinical presentation, the presence of underlying heart disease, and the potential for progression to more serious arrhythmias. Multidisciplinary collaboration between pediatric cardiologists, electrophysiologists, and, when appropriate, genetic counselors, is essential to optimize outcomes for pediatric patients with PVCs.

## Conclusion

PVCs in children represent a clinical conundrum that encapsulates the intersection of abnormal cardiac electrophysiology, genetic predisposition, and structural cardiac anomalies. The diversity in their presentation, from asymptomatic incidental findings to symptomatic arrhythmias, necessitates a nuanced and tailored approach to diagnosis and management. This review has traversed the epidemiological landscape, delineated the complex pathophysiological mechanisms, and outlined the structured diagnostic approach for pediatric PVCs, culminating in a multi-tiered treatment paradigm.

The cornerstone of managing PVCs in the pediatric cohort is a thorough risk assessment, balancing the potential for malignant arrhythmias against the iatrogenic risks of intervention. The spectrum of management strategies ranges from conservative lifestyle modifications and pharmacological therapies to advanced invasive procedures such as catheter ablation and ICD implantation. The selection of treatment is predicated on a comprehensive understanding of the individual patient's clinical context, underpinned by the latest evidence-based guidelines and best practice recommendations.

The future horizon of pediatric PVC management is illuminated by advances in genetic diagnostics, precision medicine, and evolving ablation technologies, promising enhanced specificity in therapeutic interventions and improved prognoses. Interdisciplinary collaboration and ongoing clinical research are imperative to refine our understanding of PVCs in children and to optimize outcomes. As we expand our knowledge base, the development of individualized care plans that are dynamic and responsive to the evolving clinical course will remain the bedrock of pediatric arrhythmia management.
